# Worms About Town: a citizen science project discovers microsporidian parasites of nematodes through environmental sampling

**DOI:** 10.1242/bio.062473

**Published:** 2026-06-08

**Authors:** Jonathan Tersigni, Edward B. James, Aaron W. Reinke

**Affiliations:** Department of Molecular Genetics, University of Toronto, Toronto, ON, Canada M5S 3G9

**Keywords:** Citizen science, Environmental sampling, Nematodes, Microsporidia, Infection, Host-parasite systems

## Abstract

Microsporidia are a diverse group of fungal parasites that infect humans and agriculturally important animals. *Caenorhabditis elegans* has emerged as a powerful host for investigating microsporidian biology, yet how ecological factors such as geography, seasonality, temporality, and sample substrate impact infection of *C. elegans* and other nematodes remains unclear. To address this, we developed a citizen scientist network, Worms About Town, to annually survey wild nematodes for microsporidia across Toronto, Canada. Here, we describe results from the first year (2024) of the project. One hundred twenty-seven environmental samples from various plant substrates were collected, 60 of which contained wild nematodes and four of which were infected with microsporidia. Two isolates of *Nematocida homosporus*, a known generalist microsporidian of nematodes, were identified. The other two microsporidia isolates belong to undescribed species of *Pancytospora* and *Enteropsectra*, genera distantly related to human-infecting microsporidia. Three nematode isolates of the *Caenorhabditis* genus were discovered, including two isolates of *C. elegans*, a species not previously reported in Ontario. Both *N. homosporus* isolates were found in *C. elegans*, and one can infect a laboratory strain of *C. elegans*. Our study expands knowledge of natural nematode-microsporidia diversity and provides a framework for systematically investigating how ecological factors shape this host-parasite system.

## Teaser

**Summary:** We developed a citizen scientist network to annually sample wild nematodes and their natural microsporidia parasites to better understand the natural diversity and ecology of this host-parasite system.

## INTRODUCTION

Microsporidia are obligate intracellular parasites capable of infecting many types of animals (Murareanu et al., 2021; Ruan et al., 2021). Microsporidia infection is typically detrimental for the host and economically, ecologically, and agriculturally important animals including pollinators (Lang et al., 2023) and farmed animals such as livestock, fish, and shrimp (Aranguren Caro et al., 2021; Ruan et al., 2021; Moratal et al., 2023). Several microsporidia species infect humans, and infection is more prevalent and severe in individuals with weakened immune systems (Han and Weiss, 2018; Han et al., 2021). Microsporidiosis in humans is broadly treated with the same two drugs, fumagillin and albendazole, which exhibit host toxicity by targeting essential, highly conserved eukaryotic proteins (Han and Weiss, 2018). More so, there is evidence that fumagillin, which has been historically used to control microsporidia infections in honeybees used in apiculture, is not as effective against the newly emerging honeybee parasite *Vairimorpha ceranae* (Huang et al., 2013; Biganski et al., 2024). Therefore, there is a need to better understand the total diversity of microsporidia parasites and the interactions with their hosts to develop more effective and specific treatments.

Although approximately 1700 species of microsporidia have been reported, this is likely a large underestimate of the true number given the consistent discovery of novel species each year (Franzen, 2008; Larsen et al., 2017; Murareanu et al., 2021). Sampling of animal hosts from the environment to identify novel microsporidian parasites has expanded our knowledge of both host and microsporidia phylgenetic diversity (Bojko et al., 2022; Willis and Reinke, 2022), suggesting that wild isolate sampling efforts present a practical avenue for better understanding these pathogens.

Microsporidian biology is difficult to study, as these parasites are transcriptionally inactive while outside their hosts and exhibit extreme genome reduction, with the smallest genomes of any known eukaryotes (Cuomo et al., 2012; Jespersen et al., 2022). Therefore, the use of model organisms has become a popular and effective method to study these enigmatic parasites. Microsporidia naturally infect zebrafish (Schuster et al., 2024), fruit flies (Franzen et al., 2005), rodents (Didier et al., 1995), and nematodes (Troemel et al., 2008) – all highly tractable and widely used model organisms in molecular biology. In particular, the nematode *Caenorhabditis elegans* has recently emerged as an effective model host. *C. elegans* and its natural microsporidian parasites have been used in screening experiments aiming to identify novel microsporidia-specific drug treatments (Murareanu et al., 2022; Huang et al., 2023), to interrogate specific and general host responses to infection (Reddy et al., 2017; Willis et al., 2021), and to reveal distinct tissue tropisms during infection by different microsporidia species (Luallen et al., 2016). On a broader level, infection screens utilizing diverse nematode hosts and microsporidian parasites have identified definite patterns of host specificity and tissue tropisms between nematode-infecting microsporidians (Zhang et al., 2016; Wadi et al., 2023).

The collection of wild nematodes and their natural microsporidian parasites has revealed an impressive diversity of this host-parasite system. *Nematocida parisii* was the first microsporidian species identified that infects *C. elegans* (Troemel et al., 2008). Since then, collections of *Caenorhabditis* and other genera of nematodes have led to the identification of several *Nematocida* species, such as *Nematocida ausubeli*, *Nematocida major* (Zhang et al., 2016), *Nematocida botruosus*, *Nematocida cider*, *Nematocida ferruginous* (Wadi et al., 2023), and *Nematocida displodere* (Luallen et al., 2016). Other nematode-infecting genera have also been identified, including *Enteropsectra* and *Pancytospora* in *Oscheius* and *Caenorhabditis* nematodes, respectively (Zhang et al., 2016). The *Enteropsectra* and *Pancytospora* isolates are particularly interesting as they, unlike *Nematocida* species, retain RNA interference (RNAi) machinery in their genomes (Wadi and Reinke, 2020) and may therefore be genetically tractable and offer a unique opportunity to genetically modify microsporidia during experimentation. Wild collections of nematodes and their microsporidian parasites have been instrumental in understanding the evolution and diversity of microsporidia, and continued sampling efforts present an opportunity to further learn about the relationship between microsporidia and their hosts.

Detailed methods and materials required to perform wild nematode isolation and microsporidia identification have been recently described, including how to isolate nematodes from the environment, screen them for microsporidia infection, molecularly determine nematode and parasite identities, and assess whether microsporidia isolates can infect *C. elegans* in the laboratory (Tamim El Jarkass and Reinke, 2024). Surveys for nematode-infecting microsporidians typically entail collection of wild nematodes from rotting plant substrates like fruits and vegetation, followed by parasite screening (Troemel et al., 2008; Zhang et al., 2016; Luallen et al., 2016; Wadi et al., 2023; van Himbeeck et al., 2025). While effective, these sampling methods are time-consuming and are limited by the number of sample collectors and availability of rotting plant substrates in the environment.

In this study, we aimed to establish an efficient wild nematode collection system to further explore the natural diversity of nematodes and their microsporidian parasites. Inspired by previous community-aided projects to examine *Caenorhabditis* genetic diversity (Crombie et al., 2024) and to identify viruses of *C. elegans* (Ni et al., 2026), we developed a citizen science project, called Worms About Town, that recruited local community members to collect wild nematodes across Toronto, Ontario, Canada. This project aimed to (1) discover, identify, and characterize nematode-infecting microsporidians; (2) determine how ecological factors such as geography and seasonality impact microsporidia infections of nematodes; and (3) establish an efficient wild nematode collection framework for continued annual sampling efforts.

## RESULTS

### Environmental sampling of wild nematodes by citizen scientists

**Fig. 1.**
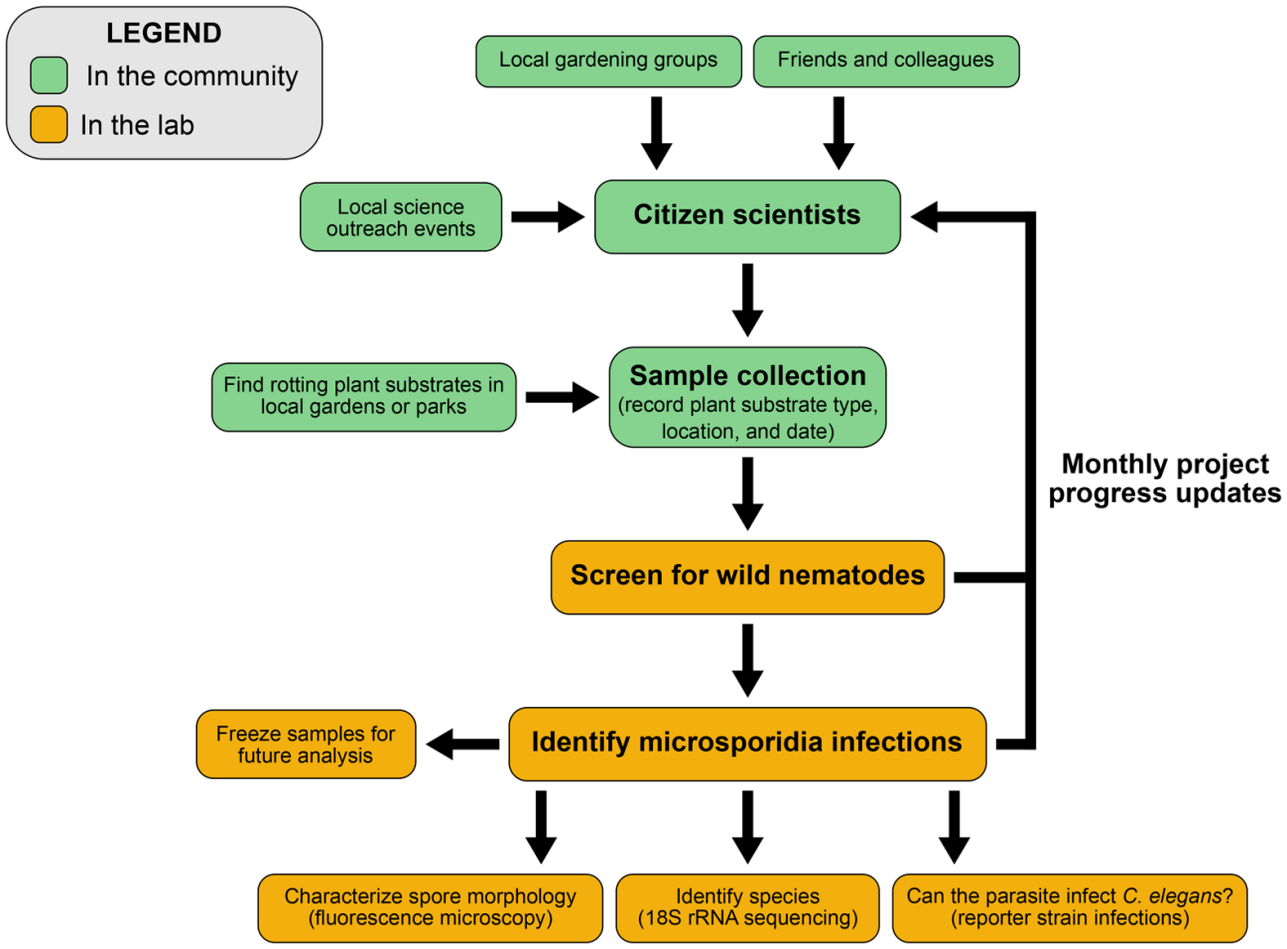
**Flowchart outlining the Worms About Town project.** Boxes in green indicate project steps involving the Toronto community (such as environmental sampling by citizen scientists), while boxes in orange indicate steps performed in the lab (such as screening samples for nematodes and microsporidia infections). Project updates and new findings were shared and discussed with all citizen scientists monthly.

**Fig. 2.**
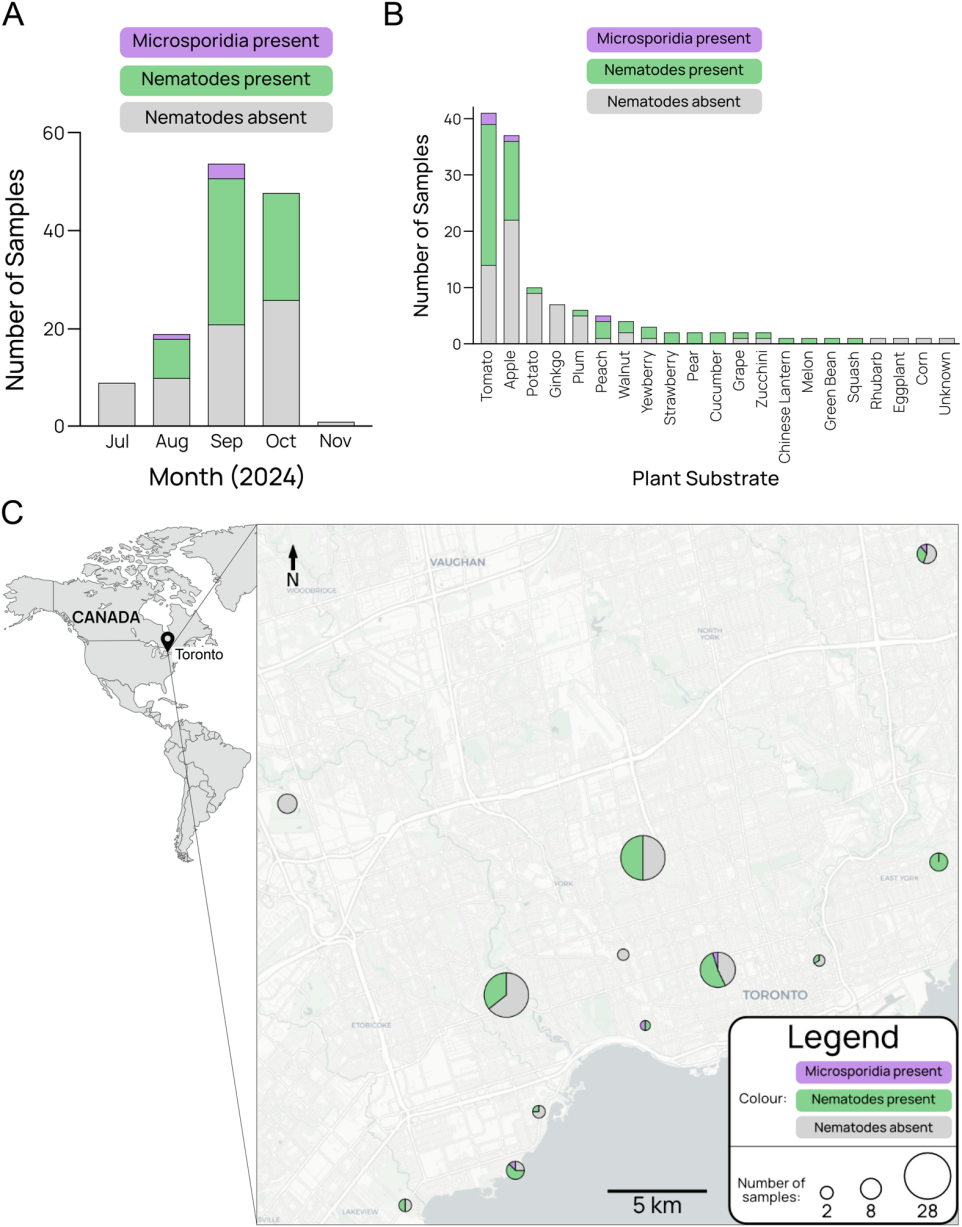
**Environmental sampling of wild nematodes and microsporidian parasites by citizen scientists.** The number of environmental samples collected in 2024 is organized by (A) month, (B) type of plant substrate, and (C) location of collection across Toronto. Gray indicates samples where no nematodes were found, while green and magenta indicate samples containing nematodes and microsporidia-infected nematodes, respectively. The pie chart size in C indicates the number of samples collected at that location, while the black line indicates a length of 5 km. Map data are copyrighted by OpenStreetMap contributors and are available from https://www.openstreetmap.org.

Citizen scientists were recruited from the Toronto community to participate in the Worms About Town project to collect wild nematodes from the environment (Fig. 1; Figs S1 and S2, Movie 1). During the first year of the project, 13 citizen scientists collected 127 total samples across Toronto, from July to November of 2024 (Fig. 2A). Almost half (*n*=60, 47%) of the samples contained nematodes, and four of the samples containing nematodes (7%) were infected with microsporidia spores (Fig. 2A). Most samples (*n*=99, 78%) were collected in September and October, and the majority of nematode-positive samples (*n*=52, 87%) were found during these months. Samples collected in July and November did not contain nematodes, although fewer total samples (*n*=10, 8%) were gathered during these months (Fig. 2A). This was due to the low abundance of rotting plant substrates in the environment, as reported to us by citizen scientists. The 127 samples were comprised of 21 types of rotten plant substrates, the most prevalent being tomato and apple (*n*=75, 59%), from which most nematode-positive samples (*n*=39, 60%) were acquired (Fig. 2B). Plant substrates were significantly associated with nematode presence or absence in the samples (Fisher's exact test with Monte Carlo simulation, *P*=0.00089). Samples with nematodes were found in 24 locations across Toronto, and all four microsporidia-positive samples came from different locations (Fig. 2C).

### Characterization of the four nematode-infecting microsporidia isolates

**Fig. 3.**
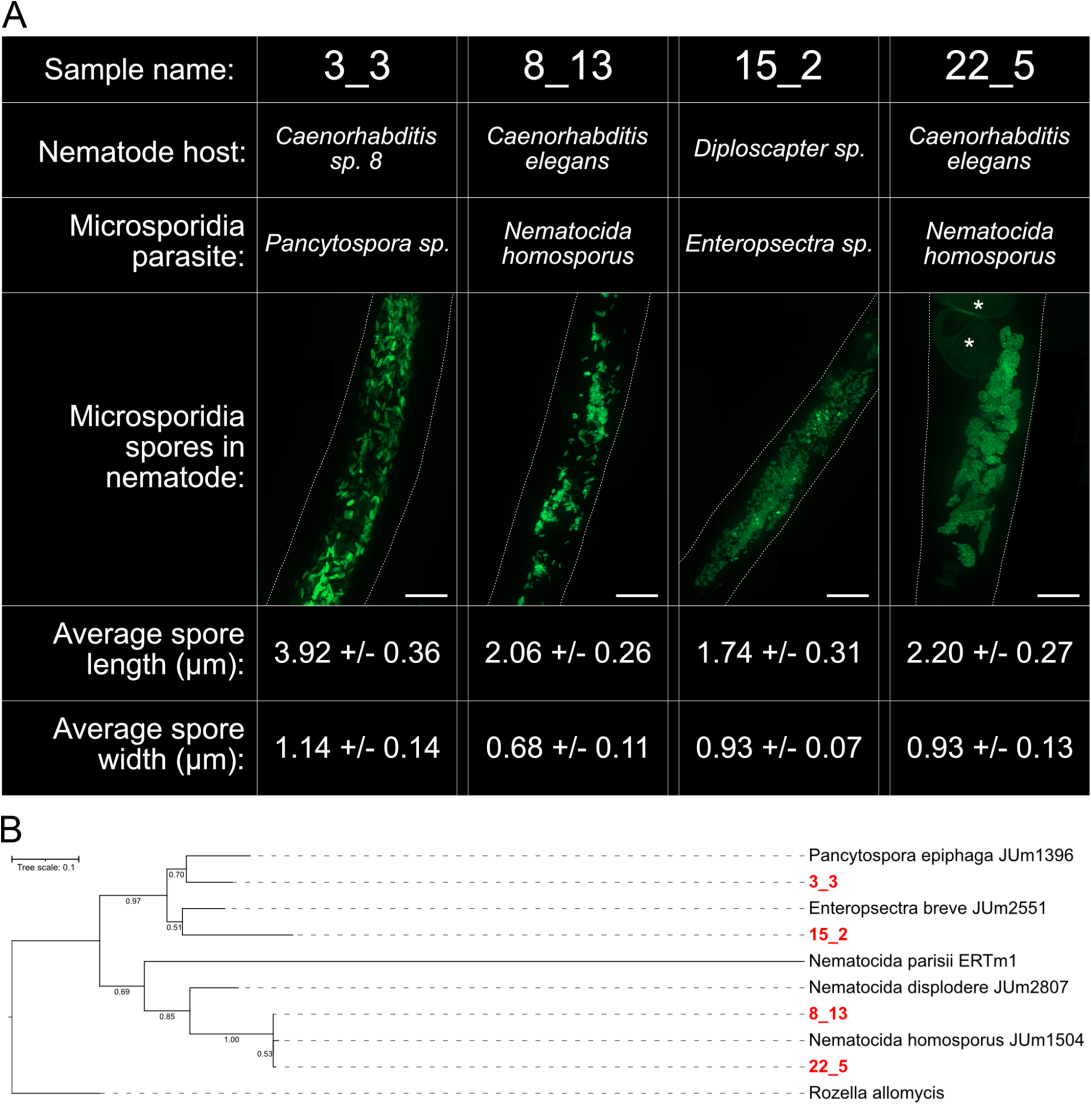
**Four nematode-infecting microsporidia isolates were discovered and characterized.** (A) Representative fluorescence microscopy images of microsporidia spores (green) stained with DY96 within a wild-caught nematode (outlined with white dashes). DY96 also stains nematode embryos, which are indicated by the asterisks in 22_5. The names of each collected sample are shown above each image, and the average spore lengths and widths are shown below in µm ± the standard deviation from the mean. Scale bars: 20 µm in length. (B) 18S rRNA phylogenetic tree of the four discovered microsporidia isolates, shown in red. Reference species, shown in black, are named followed by a reference strain identifier. The tree is rooted with the outgroup species *R. allomycis*, and the bootstrap support for 1000 tree replicates, which clustered together, are shown at each node. Scale bar shows substitutions per site.

Wild nematode isolates were stained with the fluorescent dye Direct Yellow 96 (DY96) to visualize microsporidia spores. Representative fluorescence microscopy images of spores of the four microsporidia isolates are shown in Fig. 3A. The lengths and widths of at least 20 spores from each isolate were measured, and mean values with standard deviation are displayed in Fig. 3A and Fig. S3.

To molecularly identify the four infected wild nematodes and their microsporidia parasites, we sequenced nematode (Kiontke et al., 2004) and microsporidia (Zhang et al., 2016) 18S rRNA genes (Tamim El Jarkass and Reinke, 2024). Sequence alignments of these sequences to published ones are shown in Fig. S4. The identities of the microsporidia parasites and nematode hosts are displayed in Fig. 3A. The microsporidia parasites in samples 3_3 and 15_2 were most closely related to the microsporidia genera *Pancytospora* (88.77% nucleotide identity) and *Enteropsectra* (83.83% nucleotide identity), respectively, while both the 8_13 and 22_5 parasites were identified as *Nematocida homosporus* isolates (99.31% and 100% nucleotide identity, respectively).

Nematodes from samples 3_3, 8_13, and 22_5 belonged to the *Caenorhabditis* genus (100% nucleotide identity), while 15_2 nematodes belonged to the *Diploscapter* genus (100% nucleotide identity) (Fig. S4). To determine the species of the *Caenorhabditis* nematode isolates, we amplified and sequenced the nematode ITS2 region (see Materials and Methods), which can distinguish between species of *Caenorhabditis* (Kiontke et al., 2011). Aligning these sequences to previously published ones using the Basic Local Alignment Search Tool (BLAST) determined that both the 8_13 and 22_5 nematodes were isolates of *C. elegans* (100% nucleotide identity for both), while the 3_3 nematodes were an isolate of *Caenorhabditis* sp. 8 (100% nucleotide identity) (Fig. S4). To further verify that the 8_13 and 22_5 nematodes belonged to the *C. elegans* species, we crossed them with males of a fluorescently labeled *C. elegans* strain (Au et al., 2019). Fluorescent offspring were observed from both crosses (Dataset S1), indicating that successful mating occurred and confirming that 8_13 and 22_5 nematodes were isolates of *C. elegans*.

Phylogenetic analysis of 18S rRNA sequences revealed that the microsporidia isolates 3_3 and 15_2 clustered within a clade containing a *Pancytospora* and *Enteropsectra* isolate, respectively, while both 8_13 and 22_5 microsporidia isolates clustered with a *N. homosporus* isolate (Fig. 3B).

### The *N. homosporus* isolate 22_5 can infect *C. elegans* in the laboratory

**Fig. 4.**
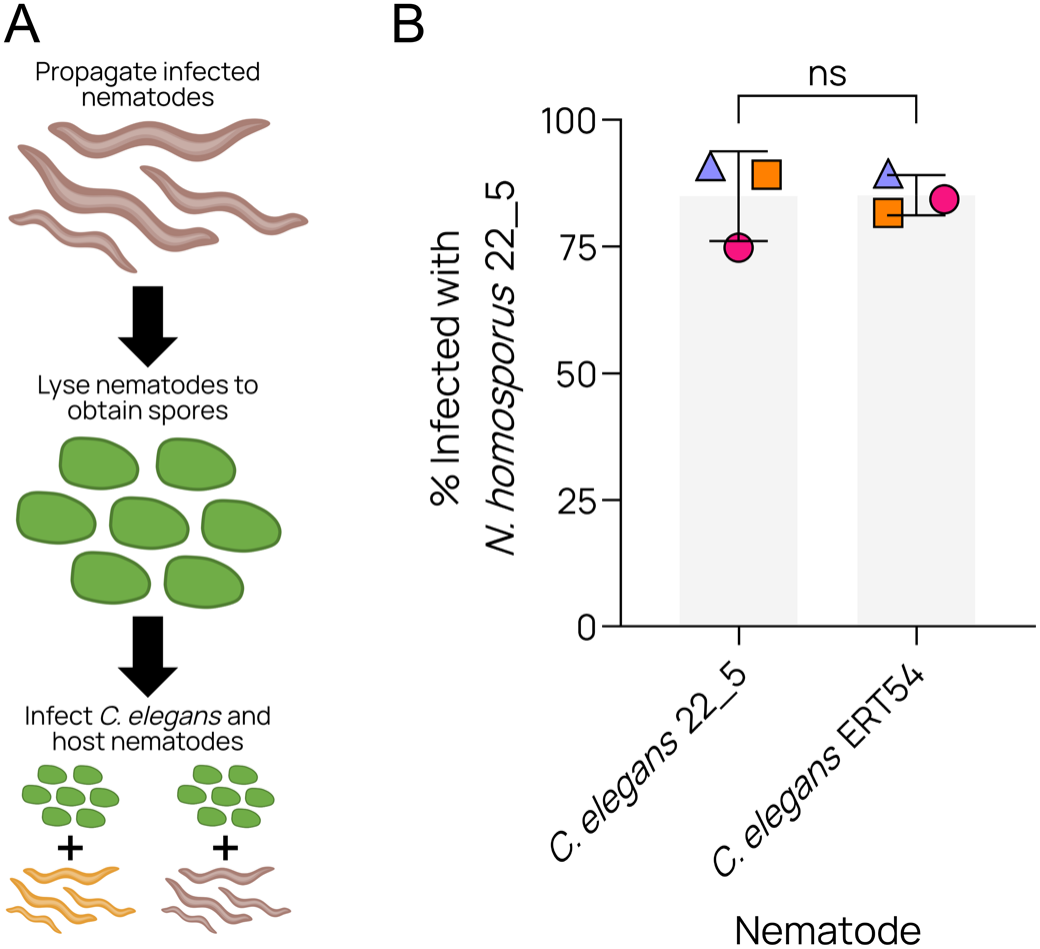
***N. homosporus* 22_5 infects a laboratory strain of *C. elegans*.** (A) Schematic depicting how microsporidia spores from infected wild nematode samples were used to infect nematodes. Infected nematodes were grown on NGM plates and then lysed to obtain concentrated microsporidia spore solutions, which were used to infect either *C. elegans* or the original host nematodes as a positive control. (B) The percentage of nematodes (*C. elegans* ERT54 or *C. elegans* 22_5) containing newly produced *N. homosporus* 22_5 microsporidia spores after 96 h post-infection. Individual biological replicates are indicated with different colors and shapes, while the mean of the three replicates is indicated with the bar. Error bars indicate the standard deviation of the mean. To determine statistical significance, a two-tailed unpaired *t*-test was performed on the data. ns, not significantly different.

Spores from the four microsporidia isolates were used to infect synchronized populations of their wild host nematodes or the *C. elegans* infection reporter strain ERT54 (Bakowski et al., 2014) (Fig. 4A). The *N. homosporus* microsporidia isolate from sample 22_5 was found to successfully infect its host nematode 22_5 (*C. elegans*) and *C. elegans* ERT54, as measured by quantifying the proportion of nematodes containing newly generated infective spores (Fig. 4B). No statistically significant difference was observed between the percentage of *C. elegans* 22_5 or *C. elegans* ERT54 nematodes infected with *N. homosporus* 22_5 spores, suggesting this parasite can infect the *C. elegans* reporter strain as successfully as the host it was isolated from. Infections by the remaining three microsporidia isolates failed to produce spores in either their respective wild nematode hosts or *C. elegans* ERT54.

## DISCUSSION

To develop a platform to annually survey wild nematodes for microsporidia infections, we developed a citizen science project called Worms About Town. We aimed to use this platform to discover new microsporidia parasites and nematode hosts, investigate ecological factors that impact infections, and maintain annual collections to study this host-parasite system over time. In the first year of the project, 13 members of the Toronto community collected 127 environmental samples, 60 of which contained wild nematodes; four nematode isolates were infected with microsporidia parasites. About 7% of the nematode-positive samples were infected with microsporidia. This microsporidia infection incidence rate is lower than the 16% reported by van Himbeeck et al. (2025) who sampled wild nematodes in The Netherlands. This observation suggests that the prevalence of nematode-infecting microsporidia in Canada may be lower than in European countries such as The Netherlands, but additional environmental sampling needs to be performed to support this claim.

In the search for wild nematodes, 21 types of plant substrates were collected. Plant substrate was found to significantly impact nematode presence or absence in the samples collected. This result suggests that certain plant substrates may better support nematode population growth and may therefore serve as microsporidia infection hubs. However, additional wild isolate collection data are needed to further analyze these patterns. Indeed, data from only 60 nematode-containing samples were used for the association between plant substrate and nematode presence, while other confounding factors such as weather at the time of sampling and size of the sampled substrate were not controlled for. These factors could have disproportionately impacted nematode prevalence in certain plant substrates. In addition to plant substrates, to investigate how geography and season impact nematode and microsporidia prevalences and how infections naturally change over time, the Worms About Town project will conduct annual environmental sampling.

From our sampling, we expanded the geographic and host diversity of nematode-infecting microsporidia species. *N*. *homosporus* was previously reported to be found in *Oscheius tipulae* nematodes in France and *Rhabditella typhae* nematodes in Portugal (Zhang et al., 2016). *N*. *homosporus* was also previously found to infect *Caenorhabditis*, including *C. elegans*, in a laboratory setting (Zhang et al., 2016)*.* Here, we report the first identification of *N. homosporus* infecting *Caenorhabditis*, specifically *C. elegans*, in the wild, and also the first identification of this microsporidia species in Canada. These results indicate that the global distribution of *N. homosporus*, and potentially other nematode-infecting microsporidia, is more extensive than previously documented. The microsporidia from samples 8_13 and 22_5 were identified as *N. homosporus* isolates using 18S rRNA sequencing, which may not be able to resolve organism identities at the species level (Reinke et al., 2017; Bojko et al., 2022). However, the spore morphologies and average spore sizes of the isolates reported here are generally consistent with those previously reported for other *N. homosporus* isolates (Zhang et al., 2016) and are within 1-10% of the length and 4-23% of the width of the average spore. *N. homosporus* is known for being a generalist parasite of multiple nematode hosts, where it infects the intestinal tract. In line with this, *N. homosporus* 22_5 was demonstrated to be able to infect a reporter strain of *C. elegans* in a laboratory setting in addition to its original host nematode. Infections by the other three discovered microsporidia isolates did not produce successful infection in the reporter strain nor their respective wild nematode hosts. This could have been due to the spore solutions used to infect nematodes not containing sufficient infective spores or the interference of infection by contaminating microbes.

We also describe novel species of *Enteropsectra* and *Pancytospora* in this study. Species of these genera are particularly interesting, as they are related to human-infecting microsporidia and, unlike *Nematocida* microsporidia, retain their RNAi machinery (Wadi and Reinke, 2020). Identifying additional isolates of these taxa that can infect *C. elegans* could present a unique opportunity to perform RNAi on microsporidia in the context of infection.

Our collection recorded the first microsporidian infection of a *Diploscapter* genus nematode – which belongs in a sister taxon to the *Caenorhabditis* genus (Kiontke and Fitch, 2005) – and two isolates of *C. elegans* nematodes. To the best of our knowledge, *C. elegans* has not previously been reported in Ontario, so our report of this species in Toronto suggests a possible range expansion (Frézal and Félix, 2015). Further collection and investigation of *C. elegans* in Ontario could reveal novel pathogens – microsporidia or others – of the nematode. Our results of *C. elegans* in Ontario could also provide an opportunity to study *C. elegans* in a new environmental context and could increase our understanding of the evolutionary history of this important genetic model organism.

The Worms About Town citizen science project will continue to perform annual sampling of wild nematodes across Toronto to further elucidate the natural diversity of this complex host-parasite system. These efforts will determine the impact of ecological factors like geography, season, and time on microsporidian infections of nematodes and may uncover novel or interesting nematode-microsporidia combinations that could be utilized in the laboratory to provide insight into mechanisms of parasite host specificity and host susceptibility/resistance to infection.

## MATERIALS AND METHODS

### Environmental sampling of wild nematodes by citizen scientists

To recruit citizen scientists for the collection of wild nematodes, we pitched the Worms About Town project to local gardening groups and at science outreach events across Toronto (Fig. 1). Here, we demonstrated how to acquire samples from rotten plant substrates, seal them in Petri dishes containing Nematode Growth Media (NGM), and screen Petri dishes for nematodes using a light microscope, which are methods that have been described recently (Tamim El Jarkass and Reinke, 2024; basic protocol 1). We also created an instructional video of these methods, which we sent to each citizen scientist who signed up for the project (Movie 1).

Nematode sample collection kits were delivered to each citizen scientist. These kits included NGM Petri dishes, ziplock bags, parafilm strips, lab gloves, an infographic brochure (Fig. S1), and a sample collection guide/tracker (Fig. S2) to record the date, location, and plant substrate collected for each sample. From July to November of 2024, citizen scientists located rotten fruit and vegetation from their personal and/or local gardens/parks and tore off small pieces of the plant substrate. Individual plant substrate samples were then placed on NGM Petri dishes for ∼24 h to allow wild nematodes to crawl onto the dish, then the plant substrate was removed from the dish. After this process, we collected the Petri dishes from the citizen scientists and screened them for the presence of nematodes using a light microscope in the lab (Fig. 1). Any nematodes discovered in the samples were then screened for microsporidia infection using the fluorescent dye DY96, which stains chitin found in microsporidia spores (Fig. 1).

### Sample nomenclature

Each citizen scientist was assigned a unique reference number, and each of their samples were sequentially numbered. For example, the sample ‘22_5’ is the fifth sample collected by the 22nd citizen scientist who signed up for the project. Some citizen scientists were assigned numbers and received collection kits but did not perform any wild nematode sampling, hence the inflated sample reference number for the 13 citizen scientists who participated in this study. Each of the four microsporidia-infected nematode isolates was assigned a designated lab strain name (AWR): 3_3 (AWR192), 8_13 (AWR193), 15_2 (AWR194), and 22_5 (AWR195).

### Microsporidia spore staining and spore size measurements

To screen wild nematodes for microsporidia infection, we followed the protocol outlined by Tamim El Jarkass and Reinke (2024). Briefly, nematodes were washed from Petri dishes with 1 ml of M9 into microcentrifuge tubes. Nematodes were pelleted by centrifuging at 1400 ***g*** for 30 s, and the supernatant was discarded. To resuspend the nematode pellet, 1 ml of acetone was used, and the nematodes were incubated at room temperature for 10 min to allow for fixation. After this period, the nematodes were again pelleted, and the supernatant was discarded. The nematodes were washed twice with 1 ml of PBS supplemented with 0.1% Tween-20 detergent. The nematodes were then resuspended with 500 µl of DY96 staining solution (1× PBS, 0.1% Tween-20, 0.1% SDS, 0.02 mg/ml DY96) and incubated on a tube rotator for 30 min covered in aluminum foil to protect them from light. The nematodes were then pelleted and resuspended with 16 µl of EverBrite Mounting Medium, which was then added to a microscope slide and covered with a coverslip. The DY96-stained nematodes were imaged on a ZEISS Axio Imager 2 at magnifications ranging from 5× to 63×. To quantify spore sizes within nematodes, a z-stack image was captured using 63× magnification, and an apotome maximum-intensity projection image was created. The lengths and widths of at least 20 randomly selected spores from each microsporidia isolate were measured from these images using the ImageJ straight-line tool (Fig. S3 and Dataset S1). Freezer stock solutions of each microsporidia-positive sample were created by washing two 10 cm plates of nematodes with 2 ml of freezing medium (see Tamim El Jarkass and Reinke, 2024; basic protocol 2) and stored at −80°C to allow for future analysis and comparison with future collections.

### 18S rRNA gene sequencing and phylogenetic analysis

To identify the nematodes and microsporidia in the four infected samples, we adapted the protocol outlined by Tamim El Jarkass and Reinke (2024). Nematodes from the four microsporidia-infected samples were continuously propagated on 10 cm NGM Petri dishes seeded with *Escherichia coli* OP50-1. To generate a DNA template for 18S rRNA PCR amplification, nematodes from two 10 cm Petri dishes per infected sample were washed into microcentrifuge tubes using 3 ml of M9. Plate washing of infected nematodes was performed instead of picking individual nematodes as described by Tamim El Jarkass and Reinke (2024; basic protocol 3) to increase the probability of obtaining infected nematodes containing microsporidia spores. The nematodes were then washed three times with M9 supplemented with 0.1% Tween-20 to remove contaminating microbes. Of the final nematode pellet, 25 µl was combined with 25 µl of single worm lysis buffer and incubated at 65°C for 60 min and then 95°C for 15 min in a thermocycler to lyse nematodes and microsporidia spores. The resulting lysate contains both nematode and microsporidia DNA and was used as a template for PCR.

The forward primer V1F (CACCAGGTTGATTCTGCCTGAC) and reverse primer 18SR1492 (GGAAACCTTGTTACGACTT) (Zhang et al., 2016) were used to amplify 22_5 and 15_2 microsporidia, while V1F and the reverse primer Micuni3R (ATTACCGCGGMTGCTGGCAC) (Doliwa et al., 2023) amplified 3_3 and 8_13 microsporidia. To amplify nematode 18S rRNA, the universal nematode forward primer G18S4a (GCTCAAGTAAAAGATTAAGCCATGC) and reverse primer DF18S-8 (GTTTACGGTCAGAACTASGGCGG) were used (Kiontke et al., 2004). To amplify the *Caenorhabditis* ITS2 region, the forward primer 5.8S-1 (CTGCGTTACTTACCACGAATTGCARAC) and reverse primer KK28S-4 (GCGGTATTTGCTACTACCAYYAMGATCTGC) were used (Kiontke et al., 2011). Sanger sequencing of the PCR products was performed, and regions of poor quality within each sequence were trimmed to generate a high-quality sequence. These sequences were used as queries for nucleotide BLAST searches to ascertain nematode and microsporidia species identities (Tamim El Jarkass and Reinke, 2024; basic protocol 3).

A maximum-likelihood phylogeny was generated using our sequenced 18S rRNA genes and reference microsporidia 18S rRNA sequences from a previous study (Zhang et al., 2016). Sequences were trimmed to exclude ambiguous bases before alignment via MUSCLE (Edgar, 2004), and tree construction was done using MEGA12 (Kumar et al., 2024). Model selection via MEGA12 suggested the Tamura model of nucleotide substitution (Tamura, 1992) with 22.59% of sites treated as evolutionarily invariable (‘T92+I’ model). We used a partial deletion cut-off of 80% to focus on near-ubiquitous sequences, reducing our dataset to 335 positions, and performed bootstrapping 1000 times. The tree was then manually rooted on the microsporidia outgroup *Rozella allomycis*.

### Wild nematode mating with a *C. elegans* GFP reporter strain

To confirm that the 8_13 and 22_5 wild nematodes are *C. elegans* isolates, we mated them with the *C. elegans* strain RG3405, which contains a GFP reporter (Au et al., 2019). As a negative control, we also included 3_3 nematodes, which from our 18S rRNA analysis were found to belong to the *Caenorhabditis* genus (*Caenorhabditis* sp. 8) but not *C. elegans* (see Results). Three larval stage 4 (L4) hermaphrodites/females of each wild nematode isolate, and five L4 RG3405 males were placed on an individual 6 cm NGM Petri dish seeded with *E. coli* OP50-1 for 24 h for mating. After the mating period, each of the three hermaphrodites/females was transferred to a fresh 6 cm NGM OP50-1 dish. Two days after this transfer, the number of GFP-positive and GFP-negative fluorescent offspring was counted on the dish. More than 100 offspring were quantified per dish, where possible (Dataset S1).

### Infection of *C. elegans* by environmental microsporidia

To generate concentrated microsporidia spore solutions of microsporidia parasites from each infected nematode sample, nematodes from three densely populated 10 cm NGM Petri dishes (approximately 2500 nematodes per dish) for each sample were resuspended with 2 ml of sterile Millipore water into a 2 ml microcentrifuge tube. The tubes were spun at 1400 ***g*** for 1 min to pellet nematodes, the supernatant was discarded, and the nematode pellets were resuspended with 1 ml of water. Two more washes were performed to dilute contaminating microbes. A final 1 ml volume of water was used to resuspend the nematode pellets, to which approximately 500 µl of 2 mm zirconia silica disruption beads was added. The tubes were vortexed in a bead disruptor at 5000 rpm for 3 min to lyse nematodes. The lysate was passed through a 5.0 µm sterile syringe filter to remove debris from the spore solution. The filter was then washed with 0.5 ml of water to gather spores remaining on the filter. The filtered spore solution was aliquoted into 50 µl volumes in 1.7 ml microcentrifuge tubes and stored at −80°C.

To determine if the wild microsporidia isolates could infect a lab strain of *C. elegans*, 50 µl of spore solution derived from each sample was added to 350 µl of sterile M9. These solutions were then used to resuspend a pellet of 1200 bleach-synchronized L1s of *C. elegans* ERT54 or one of the four wild nematode hosts. The mixtures were individually plated on 6 cm *E. coli* OP50-1-seeded NGM Petri dishes, dried in a clean cabinet, and stored at 21°C. Ninety-six hours after plating, nematodes were washed off the dishes with M9, fixed with acetone, and stained with DY96 as previously described to discern newly produced microsporidia spores.

### Statistical significance analysis

To determine whether there was a significant association between plant substrate and nematode presence or absence in the samples, we performed a Fisher's exact test (21 × 2 contingency table) with Monte Carlo simulation on the data. To determine whether there was a significant difference between the proportion of *C. elegans* 22_5 or *C. elegans* ERT54 nematodes infected with *N. homosporus* 22_5, we performed a two-tailed unpaired *t*-test on the data.

## Supplementary Material



10.1242/biolopen.062473_sup1 Supplementary information

Dataset 1. Excel spreadsheet containing all environmental sampling and experimental data from the study.
