# Supplementary material for: Worms About Town: a citizen science project discovers microsporidian parasites of nematodes through environmental sampling
Source: Biol Open. 2026 Jun 8;15(6):bio062473. doi: 10.1242/bio.062473 (PMC13312921; doi:10.1242/bio.062473)
Supplement: Supplementary information [file biolopen-15-062473-s1.pdf]

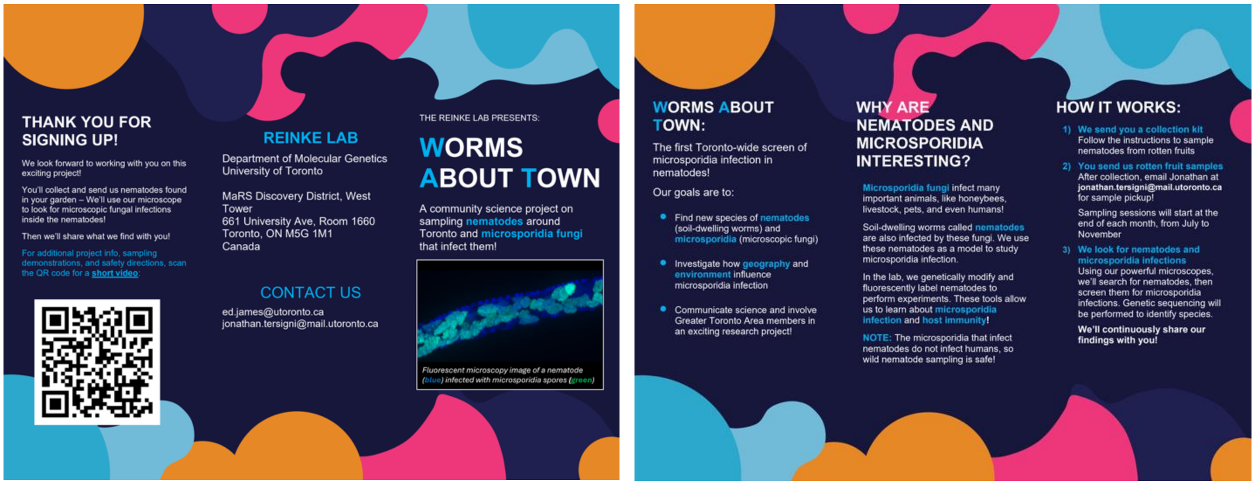

Fig. S1. Trifold brochure detailing the Worms About Town project. This document was printed double-sided and included in the nematode collection kit distributed to all citizen scientists. This document is available on the Reinke lab website (https://www.reinkelab.org/worms-about-town).

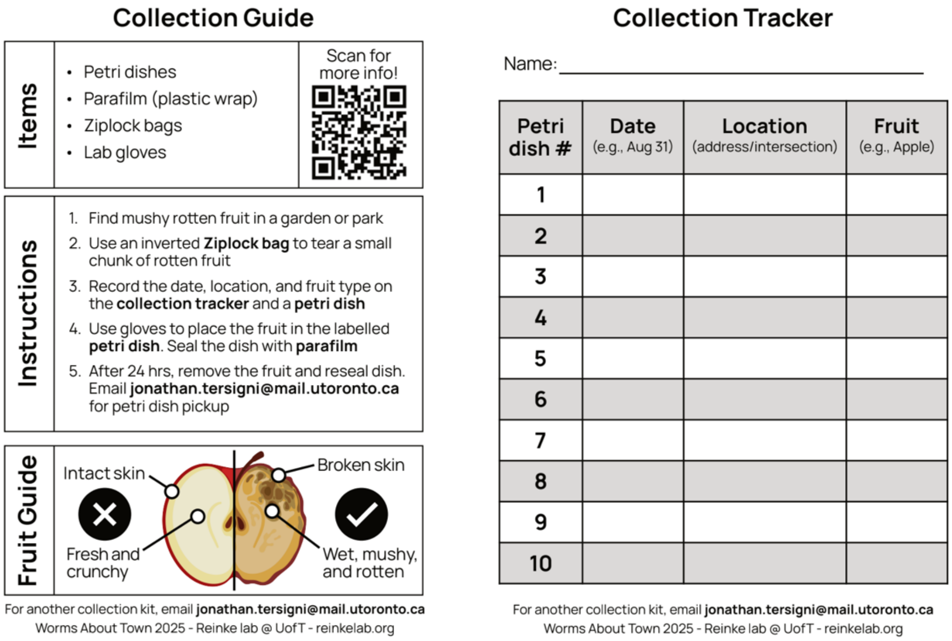

Fig. S2. Nematode sample collection guide and tracker. This document was printed double-sided and included in the nematode collection kit distributed to all citizen scientists. Citizen scientists recorded information for 10 collected samples in the Collection Tracker sheet, which was returned during sample pick-up. This document is available on the Reinke lab website (https://www.reinkelab.org/worms-about-town).

Biology Open • Supplementary information

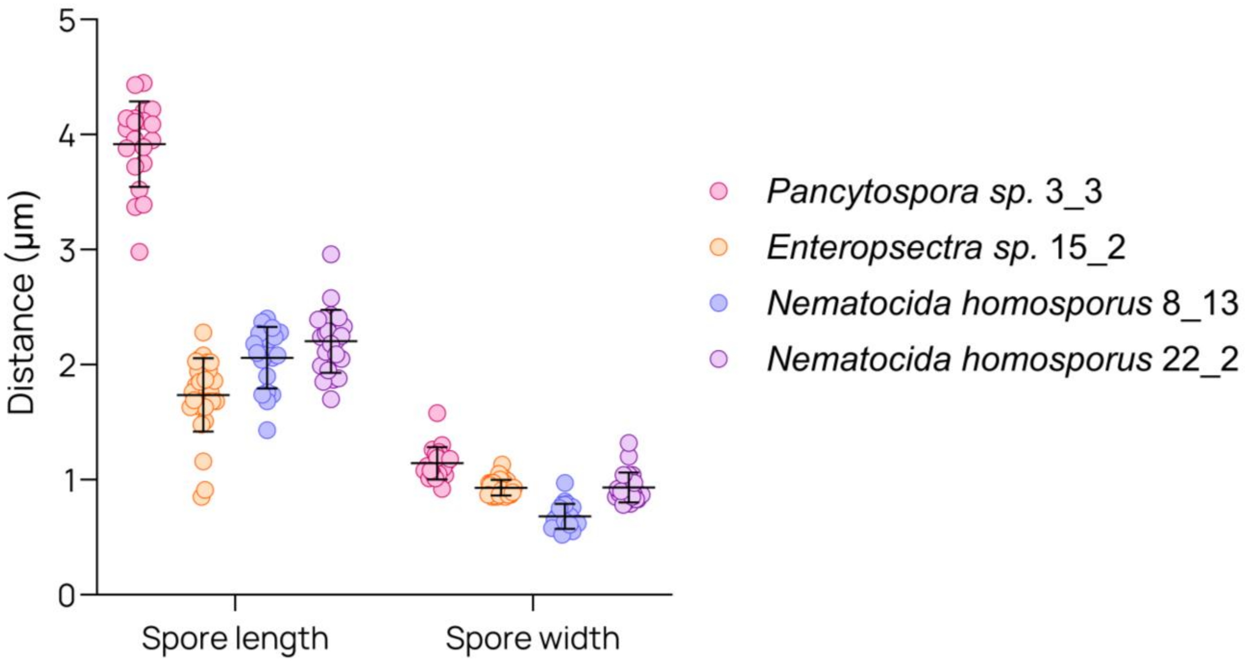

Fig. S3. Lengths and widths of individual spores from the microsporidia isolates discovered in this study. At least 20 spores were measured per isolate. Data points represent measurements from individual spores and colour indicates microsporidia isolate identity. Horizontal bars indicate mean and error bars indicate standard deviation of the data.

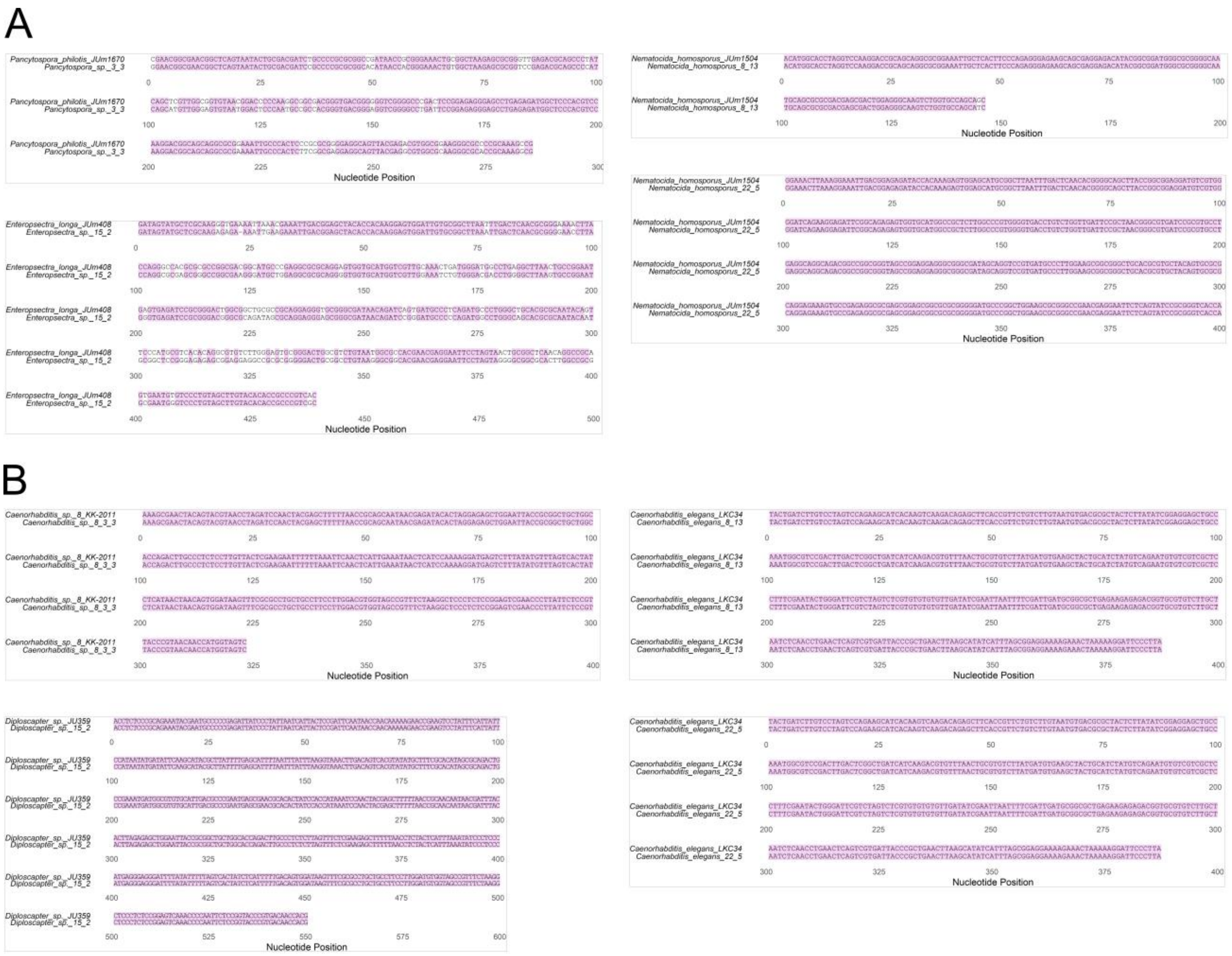

Fig. S4. Sequence alignments of microsporidia and nematode 18S rRNA genes and *Caenorhabditis* ITS2 regions. **A**) The 18S rRNA genes from the four microsporidia isolates were sequenced and aligned to the closest BLAST hit. The percent nucleotide identity between *Pancytospora sp.* 3_3 and *Pancytospora philotis* Jum1670 was 88.77% (top left); *Enteropsectra sp.* 15_2 and *Enteropsectra longa* Jum408 was 83.83% (bottom left); *Nematocida homosporus* 8_13 and *Nematocida homosporus* Jum1504 was 99.31% (top right); and *Nematocida homosporus* 22_5 and *Nematocida homosporus* Jum1504 was 100% (bottom right). **B)**The 18S rRNA gene from the isolate 15_2 or the *Caenorhabditis* ITS2 regions from the isolates_3, 8_13, and 22_5 were sequenced and aligned to the closest BLAST hit. The percent nucleotide identities between *Caenorhabditis sp. 8* 3_3 and *Caenorhabditis sp. 8* (top left), *Diploscapter sp.* 15_2 and *Diploscapter sp.* JU359 (bottom left), *Caenorhabditis elegans* 8_13 and *Caenorhabditis elegans* LKC34 (top right), and *Caenorhabditis elegans* 22_5 and *Caenorhabditis elegans* LKC34 (bottom right) were all 100%. Alignments were performed using Clustal Omega. Nucleotides highlighted in pink represent matches.

Dataset 1. Excel spreadsheet containing all environmental sampling and experimental data from the study.

Available for download at
https://journals.biologists.com/bio/article-lookup/doi/10.1242/bio.062473#supplementary-data

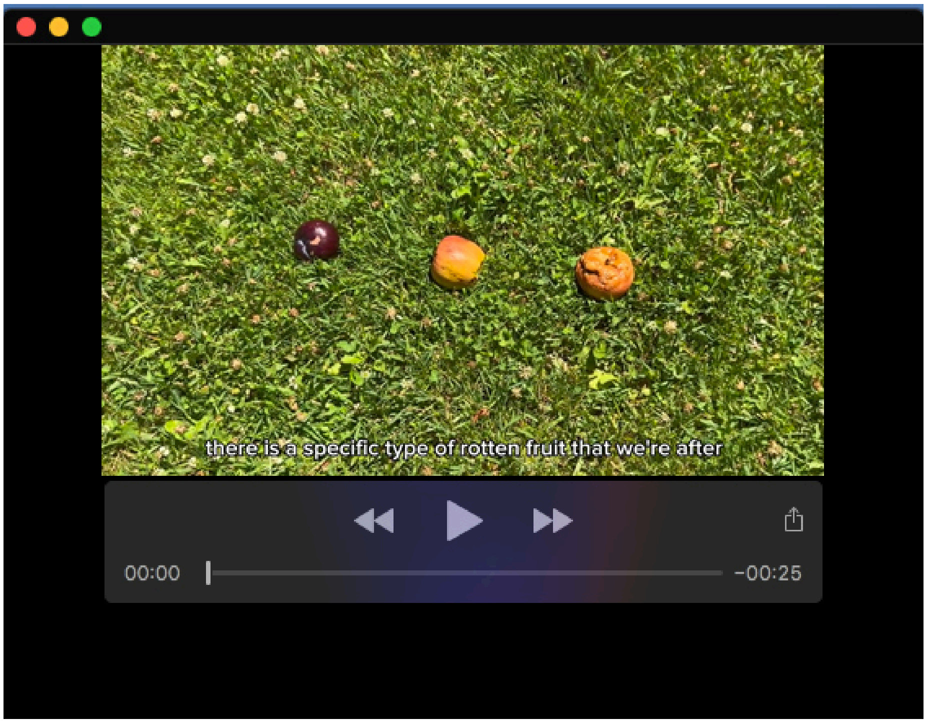

Movie 1. Instructional video that discusses the Worms About Town project and demonstrates how to collect environmental samples.

Biology Open • Supplementary information
