# Supplementary material for: Worms About Town: a citizen science project discovers microsporidian parasites of nematodes through environmental sampling
Source: Biol Open. 2026 Jun 8;15(6):bio062473. doi: 10.1242/bio.062473 (PMC13312921; doi:10.1242/bio.062473)
Supplement: Supplementary file 1 [file biolopen-15-062473_review_history.pdf]

Worms About Town: a citizen science project discovers microsporidian parasites of nematodes through environmental sampling

Jonathan Tersigni, Edward B. James and Aaron W. Reinke
10.1242/bio.062473

Editor: Sandhya Koushika

Review timeline

Original submission:	7 January 2026
Editorial decision:	19 January 2026
First revision received:	19 April 2026
Editorial decision:	29 April 2026
Second revision received:	11 May 2026
Accepted:	13 May 2026

Original submission

First decision letter

MS ID#: bio.062473

MS Title: Worms About Town: a citizen science project discovers microsporidian parasites of nematodes through environmental sampling

Authors: Jonathan Tersigni, Edward B. James and Aaron W. Reinke

I have now reached a decision on the above manuscript.

The reviewer reports are shown at the bottom of this email.

As you will see, the reviewers raised a number of substantial criticisms that prevent me from accepting the paper at this stage. Please do add the statistical tests.

They suggest, however, that a revised version might prove acceptable, if you can address their concerns. If you think that you can deal satisfactorily with the criticisms on revision, I would be pleased to see a revised manuscript. We would then return it to the reviewers.

At this stage, we also ask you to ensure your manuscript complies with our formatting guidelines. Provided you are able to fully address the referees' comments, we are positive about publication of your paper (we accept over 95% of revision submissions) and therefore hope you won't mind any extra work involved in reformatting your manuscript at this point.

Please upload both a 'clean' version of your Word file, along with a highlighted version clearly showing where you have made changes in the revised manuscript. Please avoid using 'Track changes' in Word files as these are lost in PDF conversion.

I should be grateful if you would also provide a point-by-point response detailing how you have dealt with the points raised by the reviewers in the 'Response to Reviewers' box. Please attend to all of the reviewers' comments. If you do not agree with any of their criticisms or suggestions please explain clearly why this is so.

© 2026. Published by The Company of Biologists under the terms of the Creative Commons Attribution License (https://creativecommons.org/licenses/by/4.0/).

Reviewer 1

Comments for the author

This study presents results from a survey of wild nematodes and the prevalence of microsporidia infections. The survey was conducted across the city of Toronto, utilising a network of citizen-scientists who collected rotting vegetable matter. In general, the study has some interesting results, but the manuscript requires some work.

The abstract (and the whole manuscript) should have less of a focus on the citizen-science project, as citizen-science in itself is a well-established method to collect data, and presumably wasn't the motivation behind this study. There are also enough results for this to be more than just a proof-of-concept study. The authors have outlined more biological rationale behind the study in the introduction (lines 58-60), so I request that the abstract is rewritten in more of a traditional way to cover the background around parasitic infections in nematodes, the goal of this study (outlined in lines 93-97), what was done (here can mention the use of citizen science), the results and then the interpretation of these results in the broader biological context.

The discussion is far too short; it only includes a paragraph restating results, a paragraph with a few interesting results that have become somewhat hidden, and a paragraph on the future plans for data collection. There is not enough interpretation of the findings, or any discussion of the limitations in the methods used (both in citizen science and also the lab protocols).

Please find below both a review following the rubric, and also specific line comments.

Rubric:

1. Experimental quality	
a. Does each figure have the proper controls?	Yes.
b. Are experiments performed using appropriate methods that will answer the question (or test the hypothesis or support the observations) posed by the authors? Is the right tool used for the job?	Yes.
c. Were the data analysed using appropriate statistical tests?	No. A simple model to look at whether results are significant is required (e.g. prevalence of nematodes in different plants, spatiotemporal trends). Furthermore, in the methods, there is no mention of statistical testing, but results of a two-tailed t-test are presented in Figure 4.
2. Reproducibility	
a. Were experiments in each figure performed using adequate number of biological replicates?	Yes.
b. Is there sufficient raw data to assess the rigor of the analysis?	Yes.
c. Does the methods section provide sufficient detail to permit reproducibility?	Yes.
3. Completeness	
a. Are the author's conclusions supported by the data?	Yes.

© 2026. Published by The Company of Biologists under the terms of the Creative Commons Attribution License (https://creativecommons.org/licenses/by/4.0/).

b. Are there any flaws in the experimental design that invalidate the approach taken by the authors?	No.
c. Are there experiments that have not been performed, but if true would disprove the conclusion? If yes, and if such experiments would be costly or time-consuming to perform, do the authors acknowledge this in a discussion of the limitations?	No.
4. Scholarship	
a. Do the authors cite and discuss the merits of relevant data that would argue against their conclusion?	No. There is very little discussion of the results and how they fit with the wider literature.
b. Do the authors cite and discuss the merits of relevant data that would support their conclusion?	No. There is no discussion of other studies in relation to their conclusions.
c. For techniques/methods manuscripts, Do the authors cite and discuss the current state of the field and clearly explain how the method improves the field?	This is presented as a research article, but the abstract is written as a methods manuscript. Unless the abstract changes (as suggested in the review), there is not enough context as to how this method of data collection improves the field.

Line comments:

Line	Comment
17-21	Citizen science is the method of data collection, but in itself is not a novel technique so should not be the main focus of the abstract.
42	The introduction includes interesting background information, but at times it is not clear how each paragraph relates to the aims of the study.
47-49	It is not immediately clear why this is the case. Is this your justification for more sampling? If so, then you need to explicitly state that. Because as it is, the following sentence implies that the level of sampling is sufficient.
49	Sampling efforts – such as?
56	Treated – in humans, livestock, both?
58-60	This is a good justification for the study and should be key in the abstract.
70	Check referencing style (parentheses, semi-colons).
73-74	Referencing style again.
74-75	This sentence is awkward. Have they been well described? What in short do they involve?
84-87	This is a good point, but the paragraph does not lead up to it.
88-89	If this work has been done before, then what in your study is novel? You need to state explicitly what your study adds to existing work.
97-98	This is not relevant here. There needs to be enough results for a publication (which I think you have) independent of how far along a project you are. Perhaps in the discussion you can justify what can be achieved through further data collection and say that the project is ongoing.
100	The results section contains a lot of text which is unnecessarily repeated from the methods and should be removed. It also includes interpretation and contextualisation of the results in the literature which should be moved to the discussion section.

© 2026. Published by The Company of Biologists under the terms of the Creative Commons Attribution License (https://creativecommons.org/licenses/by/4.0/).

102-103	Check the journal style – it is unusual to have a result presented in the subheading. It is also too long.
104-119	This is all methods and doesn't belong in the results section.
123	Most – be specific, how many? What percentage?
124	Majority – again, be specific.
126-127	Effort in data collection is not clear. For example, authors state that there were markedly fewer samples in July and November, but it is not clear whether this is because there was no effort or if field workers could not find rotting plant substrates (important difference).
127-129	Is this statistically significant?
131	Need to state the success rates (numbers).
132-134	This is not a result and needs to be moved to the discussion section.
132	Our lab – awkward phrase, keep ownership out of it and just reference the previous peer-reviewed work.
137-142	Methods.
144-148	Methods.
153-157	Not a result in this study, move to discussion section.
158	Belong – change to past tense and keep consistent throughout.
170-174	Methods.
179-184	Methods.
206-212	These are key interesting results and should be stated further up in the discussion section and should be discussed more thoroughly.
228	The wording makes it unclear whether the authors or the participants did this.
235	Whereupon we collected – presumably not immediately after 24 hours?
235-236	A reference here for the standard methodology would be helpful.
237	Keep wording consistent throughout – citizen scientists, volunteers, participants.
263	How were the spores selected? Randomly? And you need to acknowledge how much the sample size differed between them. In the results you present means so you also need to present standard deviation or some other measurement of uncertainty.
270	Adapted – if you state adapted, then you need to give the methods in full (not briefly as stated below). Either that, or you should say exactly what you did differently from the 2024 study. Why did they need adapted? What are the implications of this?
296	Reference to previous studies (plural) but then you only give one study.
Figure 2c	This map is too small to read. I recommend making it its own figure. It needs enlarging and it would also be helpful to a non-North American audience to include a zoomed out inset to show where Toronto is.
Figure 3a	It is not clear (and not very scientific) to state "3.9 by 1.1" to represent a length of 3.9 and a width of 1.1 – needs rewording.
Figure 4b	This plot is difficult to read – suggest either using a y-axis break at ~60% or (more importantly) jittering the datapoints for each nematode so that they are not on top of each other.
407-408	This is the only mention of this test – needs to be included in the methods and results sections.

Reviewer 2

Comments for the author

© 2026. Published by The Company of Biologists under the terms of the Creative Commons Attribution License (https://creativecommons.org/licenses/by/4.0/).

This manuscript reports the first-year outcomes of the Worms About Town citizen-science initiative, which engages community members in environmental sampling to identify wild nematodes harboring natural microsporidia infections. From 127 samples collected across Toronto, the authors identified four samples containing microsporidia-infected nematodes. These microsporidia-positive samples were predominantly recovered from rotten tomato and apple substrates collected during September and October at multiple locations.
The infected nematode isolates belonged to the genera Caenorhabditis (three samples) and Diploscapter (one sample). The associated microsporidia included two isolates of Nematocida homosporus and two previously undescribed species belonging to the Pancytospora and Enteropsectra genera. Notably, the study reports the first detection of C. elegans in Ontario and demonstrates that an environmentally isolated N. homosporus strain is capable of infecting laboratory C. elegans. Overall, this work establishes a feasible and thoughtfully designed citizen-science framework. However, the authors should address the following concerns to make their findings more convincing and conclusive.
Comments:
Figure 2C: The purple symbols and circles are too small to clearly convey that microsporidia-positive samples originate from distinct locations. Increasing symbol size or contrast would improve clarity and interpretability.
Lines 141-143: Microsporidia spores are classified based on length and width measurements; however, it is unclear how many spores were quantified and whether the reported values represent means. Please specify the exact number of spores measured. In addition, a graphical representation (e.g., scatter plot or distribution) of spore length and width would more clearly illustrate shape and morphological variation.
Lines 144-153 and 158-165: Sequence alignment data for both microsporidia and nematode identification are not shown. Please include these alignments as supplementary material to support the taxonomic assignments.
Lines 179-187: The authors demonstrate that N. homosporus isolate 22_5 can infect a laboratory C. elegans strain. It would be useful to clarify whether pathogenicity or infectivity assays were also attempted for the Pancytospora and Enteropsectra isolates, or to explicitly state if this was not tested.

Reviewer's Responses to Questions

Experimental quality
Does each figure have the proper controls?
If 'No', please indicate reasons in Comments for Author box below.
Reviewer #1:
- Yes

Reviewer #2:
- Yes

Were the data analyzed using appropriate statistical tests?
If 'No', please indicate reasons in Comments for Author box below.
Reviewer #1:
- No

Reviewer #2:
- No

Reproducibility
Were experiments performed using adequate number of biological replicates?
If 'No', please indicate reasons in Comments for Author box below.
Reviewer #1:
- Yes

Reviewer #2:

© 2026. Published by The Company of Biologists under the terms of the Creative Commons Attribution License (https://creativecommons.org/licenses/by/4.0/).

- Yes

Does the methods section provide sufficient detail to permit reproducibility?
If 'No', please indicate reasons in Comments for Author box below.
Reviewer #1:

- Yes

Reviewer #2:

- Yes

Completeness
Are the manuscript's conclusions supported by the data?
If 'No', please indicate reasons in Comments for Author box below.
Reviewer #1:

- Yes

Reviewer #2:

- Yes

Scholarship
Do the authors cite and discuss the merits of data that would argue for and against their conclusion?
If 'No', please indicate reasons in Comments for Author box below.
Reviewer #1:

- No

Reviewer #2:

- Yes

Does the manuscript title & abstract accurately reflect the contents of the manuscript, without hyperbole?
If 'No', please indicate reasons in Comments for Author box below.
Reviewer #1:

- No

Reviewer #2:

- Yes

First revision

Author response to reviewers' comments

We thank the reviewers for their comments. We have provided a revised manuscript to address the reviewers' concerns, along with an identical manuscript with highlighted changes in blue text. Points raised by the reviewers have been incorporated into the manuscript (including several new figure panels) and we provided a point-by-point response to each of the reviewer's comments here.

Reviewer #1 (Remarks to the Author):

This study presents results from a survey of wild nematodes and the prevalence of microsporidia

© 2026. Published by The Company of Biologists under the terms of the Creative Commons Attribution License (https://creativecommons.org/licenses/by/4.0/).

infections. The survey was conducted across the city of Toronto, utilising a network of citizen-scientists who collected rotting vegetable matter. In general, the study has some interesting results, but the manuscript requires some work.

We thank the reviewer for all of their comments.

Major comments:
1. The abstract (and the whole manuscript) should have less of a focus on the citizen-science project, as citizen-science in itself is a well-established method to collect data, and presumably wasn't the motivation behind this study. There are also enough results for this to be more than just a proof-of-concept study. The authors have outlined more biological rationale behind the study in the introduction (lines 58-60), so I request that the abstract is rewritten in more of a traditional way to cover the background around parasitic infections in nematodes, the goal of this study (outlined in lines 93-97), what was done (here can mention the use of citizen science), the results and then the interpretation of these results in the broader biological context.

We have modified the abstract to focus more the biological rationale of our study which is to further investigate the diversity of nematode-infecting microsporidia and their hosts and to probe the impact of ecological factors on this host-parasite system.

2. The discussion is far too short; it only includes a paragraph restating results, a paragraph with a few interesting results that have become somewhat hidden, and a paragraph on the future plans for data collection. There is not enough interpretation of the findings, or any discussion of the limitations in the methods used (both in citizen science and also the lab protocols).

We have now provided more interpretation and discussion of our findings in the discussion section.

Reviewer #2 (Remarks to the Author):

This manuscript reports the first-year outcomes of the Worms About Town citizen-science initiative, which engages community members in environmental sampling to identify wild nematodes harboring natural microsporidia infections. From 127 samples collected across Toronto, the authors identified four samples containing microsporidia-infected nematodes. These microsporidia-positive samples were predominantly recovered from rotten tomato and apple substrates collected during September and October at multiple locations. The infected nematode isolates belonged to the genera Caenorhabditis (three samples) and Diploscapter (one sample).

The associated microsporidia included two isolates of Nematocida homosporus and two previously undescribed species belonging to the Pancytospora and Enteropsectra genera. Notably, the study reports the first detection of C. elegans in Ontario and demonstrates that an environmentally isolated N. homosporus strain is capable of infecting laboratory C. elegans. Overall, this work establishes a feasible and thoughtfully designed citizen-science framework. However, the authors should address the following concerns to make their findings more convincing and conclusive.

We thank the reviewer for all of their comments.
Major comments:
1. Figure 2C: The purple symbols and circles are too small to clearly convey that microsporidia-positive samples originate from distinct locations. Increasing symbol size or contrast would improve clarity and interpretability.

To address this issue, we have increased the total size of the map image to provide better clarity of the symbols. We refrained from increasing symbol sizes to maintain geographical accuracy of the collected samples.

2. Lines 141-143: Microsporidia spores are classified based on length and width measurements; however, it is unclear how many spores were quantified and whether the reported values represent means. Please specify the exact number of spores measured. In addition, a graphical representation (e.g., scatter plot or distribution) of spore length and width would more clearly

© 2026. Published by The Company of Biologists under the terms of the Creative Commons Attribution License (https://creativecommons.org/licenses/by/4.0/).

illustrate shape and morphological variation.

We have now specified the number of spores measured for each wild microsporidia isolate (line 141) and that these reported values represent means. We have also added Supplemental Figure S3 which shows the distribution of lengths and width values measured from individual spores.

3. Lines 144-153 and 158-165: Sequence alignment data for both microsporidia and nematode identification are not shown. Please include these alignments as supplementary material to support the taxonomic assignments.

We have now added Supplemental Figure S4 displaying sequence alignment data for both microsporidia and nematode identification.

4. Lines 179-187: The authors demonstrate that N. homosporus isolate 22_5 can infect a laboratory C. elegans strain. It would be useful to clarify whether pathogenicity or infectivity assays were also attempted for the Pancytospora and Enteropsectra isolates, or to explicitly state if this was not tested.

We have clarified in the manuscript (lines 188-192) that infectivity assays for the other three microsporidia isolates were attempted in addition to *N. homosporus* 22_5. We did not see these other isolates infecting our *C. elegans* reporter strain, but we also did not observe infection of the wild nematode strain, making it not possible to determine host specificity of these isolates.

Line Comments:

17-21: Citizen science is the method of data collection, but in itself is not a novel technique so should not be the main focus of the abstract.

While we believe that the citizen scientist framework we developed for systematically sampling wild isolates across Toronto each year is a fundamental to our previous and continued findings we agree that citizen science is not a novel technique and therefore should not be the main focus of the abstract. We have restructured the abstract to better represent the main focus and findings of the manuscript.

42: The introduction includes interesting background information, but at times it is not clear how each paragraph relates to the aims of the study.

We have restructured and reordered some sections of the introduction to create a better logical flow of information and to better provide background and rationale for our study.

47-49: It is not immediately clear why this is the case. Is this your justification for more sampling? If so, then you need to explicitly state that. Because as it is, the following sentence implies that the level of sampling is sufficient.

Yes, this is justification for additional sampling provided by our study. While the following sentence (line 49) indeed describes that past sampling efforts have advanced our knowledge on microsporidia, we don’t believe this sentence implies that the current level of sampling is sufficient. We have included some additional text on lines 51 and 54 to better explain the rationale for our study.

49: Sampling efforts – such as?

We have clarified the meaning of this sentence.

56: Treated – in humans, livestock, both?

We have clarified the meaning of this sentence.

58-60: This is a good justification for the study and should be key in the abstract.

© 2026. Published by The Company of Biologists under the terms of the Creative Commons Attribution License (https://creativecommons.org/licenses/by/4.0/).

We have included this justification in the abstract (line 18).

70: Check referencing style (parentheses, semi-colons).

We have corrected this inconsistency.

73-74: Referencing style again.

We have corrected this inconsistency.

74-75: This sentence is awkward. Have they been well described? What in short do they involve?

We have reordered the beginning of this paragraph and included more detail on these described methods.

84-87: This is a good point, but the paragraph does not lead up to it.

We have relocated this point to line 79 as this sentence better fits the paragraph on use of model hosts to study microsporidia.

88-89: If this work has been done before, then what in your study is novel? You need to state explicitly what your study adds to existing work.

We have modified these lines to include more detail on what our study adds to the existing literature.

97-98: This is not relevant here. There needs to be enough results for a publication (which I think you have) independent of how far along a project you are. Perhaps in the discussion you can justify what can be achieved through further data collection and say that the project is ongoing.

We have removed this sentence from the introduction.

100: The results section contains a lot of text which is unnecessarily repeated from the methods and should be removed. It also includes interpretation and contextualisation of the results in the literature which should be moved to the discussion section.

We have removed the indicated results sections that are repeated from the methods and have relocated the interpretation sections from the results to the discussion section.

102-103: Check the journal style – it is unusual to have a result presented in the subheading. It is also too long.

We have shortened and adjusted the subheading to not reflect a result.

104-119: This is all methods and doesn't belong in the results section.

We have relocated this paragraph to Methods.

123: Most – be specific, how many? What percentage?

We have now included the number of samples and the percentage.

124: Majority – again, be specific.

We have now included the number of samples and the percentage.

126-127: Effort in data collection is not clear. For example, authors state that there were markedly fewer samples in July and November, but it is not clear whether this is because there was no effort or if field workers could not find rotting plant substrates (important

© 2026. Published by The Company of Biologists under the terms of the Creative Commons Attribution License (https://creativecommons.org/licenses/by/4.0/).

difference).

We have made this line more clear that the participants found less rotting plant substrates during July and November despite their search efforts.

127-129: Is this statistically significant?

We performed a Fisher's Exact Test to show that plant substrate significantly influenced nematode presence or absence.

131: Need to state the success rates (numbers).

We now state the success rates.

132-134: This is not a result and needs to be moved to the discussion section.

We have moved this sentence to the discussion section.

132: Our lab – awkward phrase, keep ownership out of it and just reference the previous peer-reviewed work.

We have removed this phrase.

137-142: Methods.

We have moved these sentences to the methods section.

144-148: Methods.

We have moved these sentences to the methods section, but keep here a brief sentence to explain how we acquired the subsequent results .

153-157: Not a result in this study, move to discussion section.

We have moved this sentence to the discussion section.

158: Belong – change to past tense and keep consistent throughout.

We have corrected this error.

179-184: Methods.

We have moved these sentences to the methods section.

206-212: These are key interesting results and should be stated further up in the discussion section and should be discussed more thoroughly.

We have moved this result further up the discussion and further expand upon it.

228: The wording makes it unclear whether the authors or the participants did this.

We have provided more detail here to resolve this ambiguity.
235: Whereupon we collected – presumably not immediately after 24 hours?

We have provided more detail here to resolve this ambiguity.

235-236: A reference here for the standard methodology would be helpful.

© 2026. Published by The Company of Biologists under the terms of the Creative Commons Attribution License (https://creativecommons.org/licenses/by/4.0/).

We have included a reference that recently described detailed methods for the collection and analysis of wild nematodes and their microsporidia parasites.

237: Keep wording consistent throughout – citizen scientists, volunteers, participants.

We have changed all reference to the participants of the Worms About Town project in the manuscript to “citizen scientists”.

263: How were the spores selected? Randomly? And you need to acknowledge how much the sample size differed between them. In the results you present means so you also need to present standard deviation or some other measurement of uncertainty.

We have now indicated that Data S1 displays the average spore lengths and widths and their standard deviations for each microsporidia isolate.

270: Adapted – if you state adapted, then you need to give the methods in full (not briefly as stated below). Either that, or you should say exactly what you did differently from the 2024 study. Why did they need adapted? What are the implications of this?

We have provided more detail as to how we adapted the methods and why.

Figure 2c: This map is too small to read. I recommend making it its own figure. It needs enlarging and it would also be helpful to a non-North American audience to include a zoomed out inset to show where Toronto is.

We have significantly enlarged the map in this figure and included an inset map of North America with Canada and Toronto labelled. We decided against the map panel being its own figure because we believe the data it shows fits in logically with the other two panels of this figure.

Figure 3a: It is not clear (and not very scientific) to state “3.9 by 1.1” to represent a length of 3.9 and a width of 1.1 – needs rewording.

We have now listed both the average spore length and width separately and have included +/- standard deviation after the averages.

Figure 4b: This plot is difficult to read – suggest either using a y-axis break at ~60% or (more importantly) jittering the datapoints for each nematode so that they are not on top of each other.

We have jittered the datapoints so they are not covering each other.

407-408: This is the only mention of this test – needs to be included in the methods and results sections.

We have included this test and its significance in the results and methods sections.

Second decision letter

MS ID#: bio.062473R1

MS Title: Worms About Town: a citizen science project discovers microsporidian parasites of nematodes through environmental sampling

Authors: Jonathan Tersigni, Edward B. James and Aaron W. Reinke

I have now reached a decision on the above manuscript.

© 2026. Published by The Company of Biologists under the terms of the Creative Commons Attribution License (https://creativecommons.org/licenses/by/4.0/).

The reviewer reports are shown at the bottom of this email.

As you will see, the reviewers gave favourable reports, but raised a few points that will require amendments to your manuscript. I hope that you will be able to carry these out, because we will accept your paper after these changes.

At this stage, we also ask you to ensure your manuscript complies with our formatting guidelines – please see our manuscript preparation guidelines for details. Provided you are able to fully address the referees' comments, we are positive about publication of your paper (we accept over 95% of revision submissions) and therefore hope you won't mind any extra work involved in reformatting your manuscript at this point.

Please upload both a 'clean' version of your Word file, along with a highlighted version clearly showing where you have made changes in the revised manuscript. Please avoid using 'Track changes' in Word files as these are lost in PDF conversion.

I should be grateful if you would also provide a point-by-point response detailing how you have dealt with the points raised by the reviewers in the 'Response to Reviewers' box. Please attend to all of the reviewers' comments. If you do not agree with any of their criticisms or suggestions please explain clearly why this is so.

Reviewer 1

Comments for the author

The authors have greatly improved this manuscript. There are a few other points that would be good to consider.
Line 123: I think you just need to either present an exact percentage to multiple decimal places, or round to nearest whole percentage. I would avoid the ~ unless you are rounding to larger increments, e.g. 50%, 75%...
Line 125: include "n=99, 78%" here and similar to all subsequent n numbers.
Line 140: report which average, e.g. mean, median... You should also report standard deviation here too. If easier, this information could go in a table.
Line 183: This discussion is greatly improved. However, I think it should start by linking back to the aims of the study.
Line 192: Just a minor grammatical point, but I would avoid starting sentences with numbers. If vital, then write out the number in text form.
Line 195: I think you have to be specific here. What were you unable to control for, and what impact (if any) you think it could have on your results. You can state them here and then address them further down, or just extend this section.

Reviewer's Responses to Questions

Experimental quality

Are experiments performed using appropriate methods that will answer the question (or test the hypothesis or support the observations) posed by the authors? Is the right tool used for the job?
If 'No', please indicate reasons in Comments for Author box below.

Reviewer #1:
Yes

Reviewer #2:
Yes

Were the data analyzed using appropriate statistical tests?
If 'No', please indicate reasons in Comments for Author box below.

Reviewer #1:
Yes

© 2026. Published by The Company of Biologists under the terms of the Creative Commons Attribution License (https://creativecommons.org/licenses/by/4.0/).

Reviewer #2:
Yes

Reproducibility
Were experiments performed using adequate number of biological replicates?
If 'No', please indicate reasons in Comments for Author box below.
Reviewer #1:
Yes

Reviewer #2:
Yes

Is there sufficient raw data to assess the rigor of the analysis?
If 'No', please indicate reasons in Comments for Author box below.
Reviewer #1:
Yes

Reviewer #2:
Yes

Does the methods section provide sufficient detail to permit reproducibility?
If 'No', please indicate reasons in Comments for Author box below.
Reviewer #1:
Yes

Reviewer #2:
Yes

Completeness
Are the manuscript's conclusions supported by the data?
If 'No', please indicate reasons in Comments for Author box below.
Reviewer #1:
Yes

Reviewer #2:
Yes

Scholarship
Do the authors cite and discuss the merits of data that would argue for and against their conclusion?
If 'No', please indicate reasons in Comments for Author box below.
Reviewer #1:
Yes

Reviewer #2:
Yes

Does the manuscript title & abstract accurately reflect the contents of the manuscript, without hyperbole?
If 'No', please indicate reasons in Comments for Author box below.
Reviewer #1:
Yes

Reviewer #2:
Yes

© 2026. Published by The Company of Biologists under the terms of the Creative Commons Attribution License (https://creativecommons.org/licenses/by/4.0/).

Second revision

Author response to reviewers' comments

We thank the reviewers for their comments. We have provided a revised manuscript to address the reviewers' concerns, along with an identical manuscript with highlighted changes in blue text. Points raised by the reviewers have been incorporated into the manuscript and we provided a point-by-point response to each of the reviewer's comments here.

Reviewer #1 (Remarks to the Author):

The authors have greatly improved this manuscript. There are a few other points that would be good to consider.

We thank the reviewer for all of their comments.

Line comments:
Line 123: I think you just need to either present an exact percentage to multiple decimal places, or round to nearest whole percentage. I would avoid the ~ unless you are rounding to larger increments, e.g. 50%, 75%...

We have rounded each percentage to the nearest whole number to increase clarity.

Line 125: include "n=99, 78%" here and similar to all subsequent n numbers.

We have added "n= X" to better indicate to readers that these numbers reflect the number of samples.

Line 140: report which average, e.g. mean, median... You should also report standard deviation here too. If easier, this information could go in a table.

Instead of listing each mean value and its standard deviation in the text, we have simply referred to Figure 3 and Figure S3 for increased clarity.

Line 183: This discussion is greatly improved. However, I think it should start by linking back to the aims of the study.

We have began the discussion by referring to the aims of the study outlined in the introduction.

Line 192: Just a minor grammatical point, but I would avoid starting sentences with numbers. If vital, then write out the number in text form.

We have reordered this sentence to avoid this grammatical issue.

Line 195: I think you have to be specific here. What were you unable to control for, and what impact (if any) you think it could have on your results. You can state them here and then address them further down, or just extend this section.

We have specified confounding factors we were unable to control for and elaborated on their potential impact of the results.

Third decision letter

MS ID#: bio.062473R2

© 2026. Published by The Company of Biologists under the terms of the Creative Commons Attribution License (https://creativecommons.org/licenses/by/4.0/).

MS Title: Worms About Town: a citizen science project discovers microsporidian parasites of nematodes through environmental sampling

Authors: Jonathan Tersigni, Edward B. James and Aaron W. Reinke

I am happy to tell you that your manuscript has been accepted for publication in Biology Open, pending our standard publication integrity checks. It was accepted on 13th May 2026.

© 2026. Published by The Company of Biologists under the terms of the Creative Commons Attribution License (https://creativecommons.org/licenses/by/4.0/).
